# 5-Fluorouracil as a Tumor-Treating Field-Sensitizer in Colon Cancer Therapy

**DOI:** 10.3390/cancers11121999

**Published:** 2019-12-12

**Authors:** Yeon-Joo Lee, Jae-Min Cho, Sei Sai, Ju Yeon Oh, Ji-Ae Park, Se Jong Oh, Misun Park, Junhye Kwon, Ui Sup Shin, Jeong-Hwa Baek, Sun Ha Lim, Jie-Young Song, Sang-Gu Hwang, Eun Ho Kim

**Affiliations:** 1 Division of Radiation Biomedical Research, Korea Institute of Radiological and Medical Sciences, Seoul 01812, Korea; eyeonjoo@kirams.re.kr (Y.-J.L.); chojaemin09@yuhs.ac (J.-M.C.); immu@kirams.re.kr (J.-Y.S.); 2 Department of Basic Medical Sciences for Radiation Damages, National Institute of Radiological Sciences, Chiba 263-8555, Japan; sai.sei@qst.go.jp; 3 Laboratory of Biochemistry, School of Life Sciences and Biotechnology, Korea University, Anam-ro 145, Seongbuk-gu, Seoul 136-701, Korea; songoh10@korea.ac.kr; 4 Division of Applied RI, Korea Institute of Radiological and Medical Sciences, Seoul 01812, Korea; jpark@kirams.re.kr (J.-A.P.); osj5353@kirams.re.kr (S.J.O.); 5 Department of Radiological & Clinical Research, Korea Cancer Center Hospital, Korea Institute of Radiological and Medical Sciences, Seoul 01812, Korea; usre@kirams.re.kr (M.P.); jhkwon@kirams.re.kr (J.K.); 6 Department of Surgery, Korea Institute of Radiological and Medical Sciences, Seoul 01812, Korea; uisupshin@kirams.re.kr; 7 Radiation Biology Research Team, Research Center, Dongnam Institute of Radiological and Medical Sciences, Busan 46033, Korea; jihan918@dirams.re.kr; 8 Department of Biochemistry, School of Medicine, Daegu Catholic University, 33, 17-gil, Duryugongwon-ro, Nam-gu, Daegu 42472, Korea; sunhalimha@gmail.com

**Keywords:** tumor-treating fields, colon cancer, 5-fluorouracil

## Abstract

Colorectal cancer (CRC) is a major cause of mortality that can be treated effectively with chemotherapy and radiotherapy, although resistance to these therapeutic modalities often occurs. Tumor-treating fields (TTFields) can block tumor growth by selectively impairing tumor cell division. In this study, we investigated the mechanism by which 5-fluorouracil (5-FU) sensitizes tumor cells to TTFields. Human HCT116 and SW480 CRC cells were treated with 5-FU and/or TTFields, and characterized in vitro in terms of cell viability, apoptosis through reactive oxygen species production, autophagy, and metastatic potentials. The biological effects of 5-FU and/or TTFields were studied via positron emission tomography and computed tomography on xenograft tumor growth and were confirmed with organoid models of patients. Our results revealed that combination treatment with 5-FU and TTFields increased the efficiency of TTFields therapy in colon cancer cells by downregulating signaling pathways associated with cell proliferation, survival, cell invasion, and migration while upregulating pathways mediating apoptosis and autophagic cell death. The novel mechanistic insights gleaned in this study suggest that combination therapy with TTFields and 5-FU may be effective in treating CRC, although safety and efficacy testing in patients with CRC will need to be performed before this strategy can be implemented clinically for TTF-sensitization.

## 1. Introduction

Colorectal cancer (CRC) is ranked among the most common types of cancer and is the third leading cause of cancer-related mortality in the world [1]. Although CRC incidence rates have been declining by 3% per year in the USA, it is estimated that approximately 1.4 million cases will be newly diagnosed each year worldwide, resulting in ~774,000 deaths [2]. Currently, chemotherapy and radiotherapy are the two most effective protocols for treating CRC. Radiotherapy is a standard therapy used in the adjuvant treatment of colon and rectum cancers following resection [3], and radiotherapy in combination with chemotherapy can further reduce local failure and distant metastasis, thereby improving therapeutic results [4,5]. Adjuvant 5-fluorouracil (5-FU)-based chemotherapy has been applied as standard therapy for patients with stage-III colon cancer following resection [6]. Combination treatment with 5-FU and other chemotherapeutic agents, including leucovorin and oxaliplatin, improved disease-free survival and overall survival [7,8]. However, resistance is common during treatment and remains an unsolved problem in the clinical application [7]. Thus, new approaches to overcoming acquired resistance are needed to improve the efficiency of clinical response to therapy.

Recently, tumor-treating fields (TTFields) have shown promise in clinical protocols involving low-intensity, intermediate-frequency, alternating electric fields that are delivered through noninvasive transducer arrays put locoregionally around the tumor [8]. TTFields have received FDA approval for recurrent and newly diagnosed GBM after surgery and radiotherapy with the adjuvant, temozolomide [9,10,11]. Moreover, the most recent National Comprehensive Cancer Network guidelines recommended TTFields in newly diagnosed GBM as a category 2A treatment for patients with better application prestige [12]. Previous findings have shown that, although the clinical efficiency of TTFields alone remains contentious, combination therapy with TTFields and chemotherapy is better than chemotherapy alone for patients newly diagnosed with GBM [9]. Although these findings collectively show the efficacy of TTFields as an anticancer device, further mechanistic investigations are needed to optimize the use of TTFields in combination with additional modalities including radiation therapy. In the future, the scope of TTFields therapy will continue to extend to incorporate new and previously unapplied tumors. Recently, preclinical investigations on the use of TTFields were performed for the following cancer types: breast, cervical, colorectal, gastric, hepatocellular, melanoma, renal, urinary transitional cell, and small cell lung cancer [13].

5-FU is a pyrimidine analog and is the most widely used chemotherapeutic agent for treating various solid cancers, including CRC. Its mechanism has been identified as the production of cytotoxic metabolites that incorporate into RNA and DNA and block thymidylate synthase, finally leading to cell cycle arrest and apoptosis in cancer cells [6]. The aim of this study was to evaluate the biological activity of 5-FU in TTFields therapy as a TTFields-sensitizer. It was found to transform colon cancer cell lines, which suggests that cell signaling pathways are activated after TTFields plus 5-FU combination therapy. We suggest that combination treatment with 5-FU and TTFields impaired CRC cell proliferation, survival, cell invasion, and migration while promoting apoptosis and autophagic cell death. We hope that our observations provide important new insight and experimental evidence supporting the use of multimodal treatment of CRC with TTFields + 5-FU combination therapy as previously reported in with TTFields + sorafenib combination therapy [14].

## 2. Results

### 2.1. 5-FU Promoted TTFields-Sensitivity In Vivo

**Figure 1:**
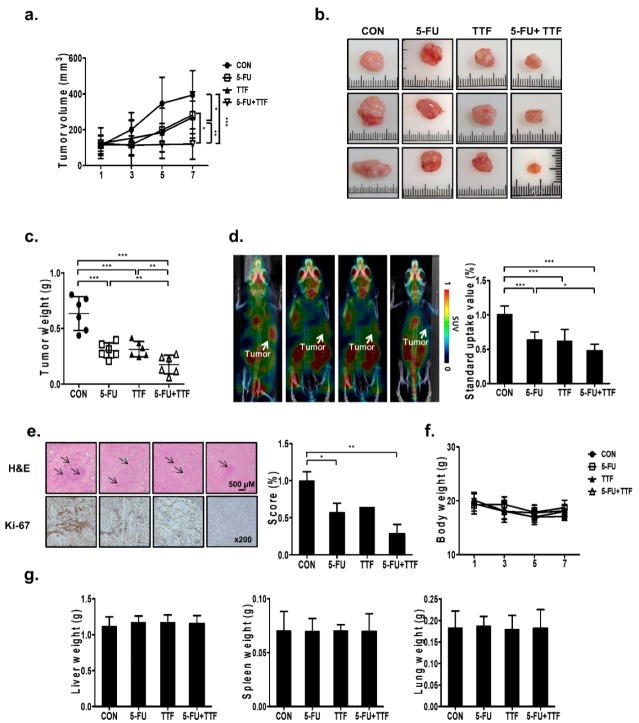
The TTFields-sensitizing effects of 5-FU on the colon, in in vivo models. (**a**) Nude mice were inoculated with HCT116 cells and treated with 5-FU or TTFields or a combination of both. Tumor volumes were measured at the indicated time points as shown using the formula: Volume = (Length × Width^2^) × 3.14/6 (*n* = 6); * *p* < 0.05, ** *p* < 0.01, *** *p* < 0.001. (**b**) Image of isolated tumors derived from control or TTFields-treated mice. bar = 1 cm. (**c**) Tumors were excised and weighed at the end of the experiment (seven days); ** *p* < 0.01, *** *p* < 0.001. (**d**) Representative positron emission tomography (PET)/computed tomography (CT) images of HCT116 tumor-bearing mice after injection of [^18^F]-FDG. The radioactivity of [^18^F]-FDG in tumors is presented as the maximal value of standard uptake values (SUV) (mean ± standard deviation (SD)); * *p* < 0.05, *** *p* < 0.001. (**e**) hematoxylin and eosin (H&E) and Ki-67 staining was conducted by immunohistochemistry, * *p* < 0.05, ** *p* < 0.01. (**f**,**g**) The liver, spleen, and lung tissues of mice were weighed at the last experiment (seven days).

To investigate the sensitizing effect of 5-FU on TTFields-mediated inhibition of tumor growth in vivo, we generated a subcutaneous model of colon cancer by injecting human HCT116 cells into nude mice. Xenografts treated with TTFields and 5-FU in combination showed a slower growth rate than the control or monotherapy groups (Figure 1a). Thus, the tumor sizes and weights after single-treatment groups were significantly larger than those in the combined treatment group (Figure 1b,c). As shown in Figure 1d, low uptake of ^18^F-fluoro-2-deoxy-d-glucose ([^18^F]-FDG) was observed in tumors treated with TTFields and 5-FU in combination, compared with that in the single-agent treatment group. The maximum SUVs were 1.01 ± 0.12 in the control group, 0.64 ± 0.11 in the 5-FU-treatment group, 0.62 ± 0.17 in the TTFields treatment group, and 0.48 ± 0.10 in the combined treatment group (Figure 1d). Single agent-treated xenografts showed stronger Ki-67 staining than those in mice subjected to combination treatment (Figure 1e).

No visible signs of toxicity were found in mice after TTFields or 5-FU treatment, as observed by the absence of morphological differences in the whole body and organs such as the liver, spleen, and lung (Figure 1f,g). The blood test results also did not show difference in the control and treatment groups (Supplementary Figure S1). These data indicated that 5-FU could enhance TTFields-sensitivity in vivo as a TTFields-sensitizer.

### 2.2. TTFields Treatment Does Not Result in Any Observable Pathologic Abnormalities in Normal Tissues

**Figure 2:**
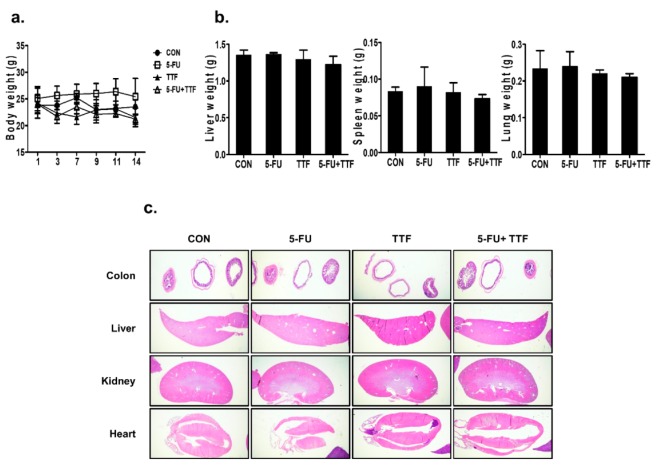
Effects of TTFields on normal tissue in mice. (**a**) The body weights of the mice were not considerably different among the 5-FU, TTFields, and combination-treated groups. (**b**) The liver, spleen, and lung tissues of the mice were weighed at the end of the experiment (14 days). (**c**) H&E staining was examined by immunohistochemistry.

To study typical tissue complications in vivo after combinatorial treatment, mice were treated with 5-FU or TTFields for 14 days without injecting tumors (Figure 2). During the treatment, the mice in the control and treatment groups showed negligible body and organ weight differences, suggesting that the combination treatment did not lead to inordinate stress in the treated mice (Figure 2a,b). The H&E staining was performed using organs obtained from the control mice, the mice treated with 5-FU or TTFields, or the combination group for 14 days (Figure 2c). Fourteen days of combinatorial treatment did not show severe pathologic abnormalities in normal tissues (Figure 2c). Collectively, the above data suggest that TTFields combined with 5-FU inhibits the growth of colon cancer in vivo without pathologic abnormalities in normal tissues.

### 2.3. TTFields-Sensitizing Events of 5-FU on In Vitro Models of Colon Cancer

**Figure 3:**
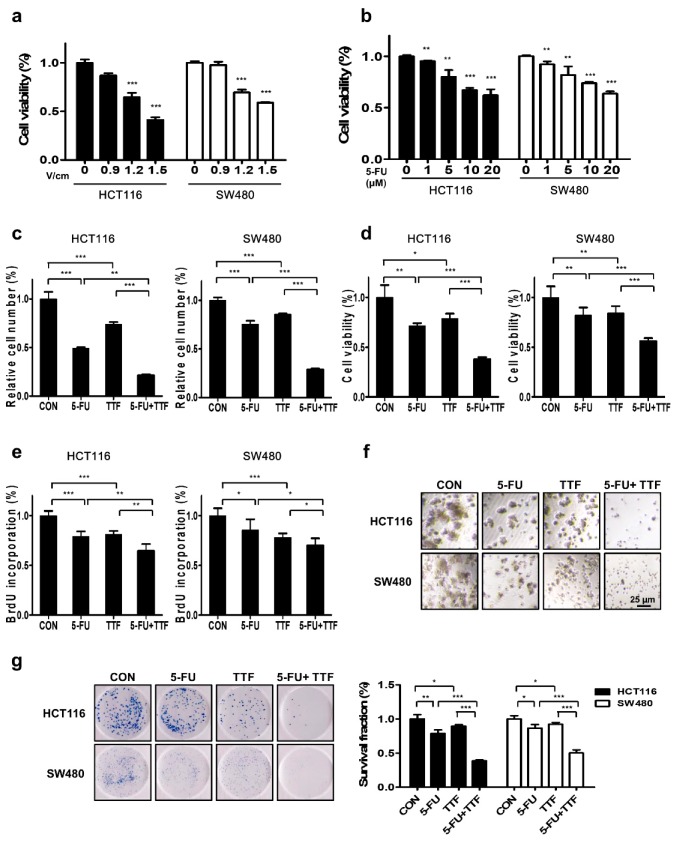
Effects of 5-FU or TTFields on cell viability in colon cancer cells. (**a**) TTFields decreased colon cell viability in an intensity-dependent manner. Cell viability was conducted by MTT assay in HCT116 and SW480 cells treated with the indicated voltages of TTFields; *** *p* < 0.001. (**b**) 5-FU inhibited colon cancer cell viability in a dose-dependent manner. Cell viability was evaluated by MTT assay in HCT116 and SW480 cells treated with the indicated doses of 5-FU; ** *p* < 0.01, *** *p* < 0.001. (**c**–**e**) the viability of cells treated with a combination of TTFields and 5-FU was considerably lower than that of cells treated with single-treatment. The proliferation rate was detected by trypan blue cell viability assay, MTT assay, and BrdU-labeling; * *p* < 0.05, ** *p* < 0.01, *** *p* < 0.001. (**f**) 3D culture model after 48 h TTFields + 5-FU treatment. (**g**) Colony-formation assays were performed using HCT116 and SW480 cells treated with the indicated treatment for 7–9 days (*n* = 3).; * *p* < 0.05, ** *p* < 0.01, *** *p* < 0.001; All values represent the means ± SD (*n* = 5).

To investigate the sensitizing effects of the combinatorial treatment in colon cancer cell lines, first of all, we applied various voltages to HCT116 and SW480 cells for 48 h to select the optimal voltage for TTFields (Figure 3a). Both colon cancer cell lines showed decreased cell viability, depending on the voltage applied, with approximately 10% viability inhibition observed at 0.9 V/cm. Next, HCT116 and SW480 cells were treated with different concentrations of 5-FU to evaluate its effects on colon cancer cells in 3-(4,5-dimethylthiazol-2-yl)-2,5-diphenyltetrazolium bromide (MTT) assays (Figure 3b). Cell growth was significantly inhibited (*p* < 0.001) after a 48-h treatment with ≥10 µM 5-FU. These data indicated that HCT116 and SW480 cells showed concentration-dependent 5-FU sensitivity.

Combination treatment with 5-FU and TTFields showed significant increase in antitumor effects in the HCT116 and SW480 cells than either treatment alone, as determined by using trypan blue cell viability assay, MTT assay, and BrdU-labeling (Figure 3c–e).

Furthermore, colonies in single-agent-treated three dimensional (3D) cultures were larger than those formed by combination treatment cells (Figure 3f). In the colony-formation assay, the survival fraction values were decreased synergistically after combination treatment with TTFields and 5-FU compared with those after single-agent treatments (Figure 3g). To determine whether TTFields has a sensitizing effect with 5-FU in HCT116 and SW480 cell lines, survival fraction was evaluated by the Valeriote and Carpentier formulas [15,16]. As shown in Table 1, each SF (surviving fraction) value presented in the combination treatment group with TTFields and 5-FU has a synergistic effect in HCT116 and SW480 cells. Lastly, we also confirmed the TTFields-sensitizing effect of 5-FU in other CRC lines to clarify its combination effects (Supplementary Figure S2). The outcomes were consistent with the results of the HCT116 and SW480 cell lines. Our results suggested that 5-FU has a TTFields-sensitizing effect on colon cancer cells in vitro.

### 2.4. Effect of TTFields on 5-FU-Induced Apoptosis

**Figure 4:**
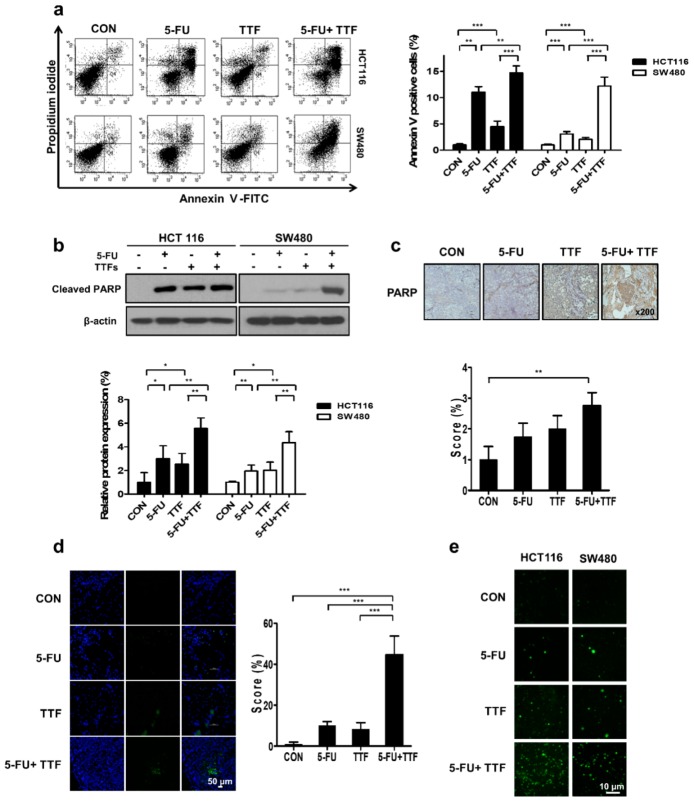
TTFields increased the apoptosis of colon cancer cells. (**a**) Annexin V and PI staining after HCT116 and SW480 cells were exposed to 6 h of 5-FU, followed by 48 h of TTFields, indicated as the TTFields, 5-FU, and TTFields + 5-FU treatments, respectively. The values represent the means of three experiments ± SD, ** *p* < 0.01, *** *p* < 0.001. (**b**) HCT116 and SW480 cells were exposed to 48 h of TTFields, 5-FU, or combination treatment. Immunoblotted (IB) cell lysates (30 μg) are shown with the indicated antibodies; The relative protein quantification for cleaved PARP normalized with β-actin. Values represent the means ± SD (*n* = 4); * *p* < 0.05, ** *p* < 0.01 (**c**) Cleaved PARP expression in the subcutaneous injection model was examined by immunohistochemistry. Representative images are provided as indicated. Values represent the means ± SD (*n* = 3); ** *p* < 0.01. (**d**) Terminal deoxynucleotide transferase-mediated dUTP nick-end labeling assays were performed using xenografts. Values represent the means ± SD (*n* = 3); *** *p* < 0.001. (**e**) HCT116 and SW480 cells were treated with TTFields, 5-FU, or the indicated combinations, and ROS levels were examined by 2′,7′-dichlorofluorescein diacetate, a peroxide-sensitive dye, during the final 30 min of incubation. Intracellular levels of ROS are shown by confocal laser fluorescence microscopy.

It is known that TTFields induces cell death by causing DNA damage [17]. To verify apoptosis, we performed flow cytometry for detecting Annexin V with a FITC-conjugated antibody and propidium iodide (PI) staining. The percentages of cell death in HCT116 and SW480 cells at 48 h after TTFields + 5-FU treatment were 14.69 and 12.18%, respectively, which were higher than those resulting from either TTFields alone (4.49 and 2.06%, respectively) or 5-FU alone (11.04 and 3.09%, respectively) (Figure 4a). These experimental results suggest that TTFields-induced apoptosis in colon cancer cells and that including a synergistic effect with 5-FU.

Our results also showed that cell death rates increased with increasing time after the TTFields application (Figure 4). We also examined whether the 5-FU-dependent enhancement of TTFields-induced cytotoxicity resulted from activation of the chief executioner of cell death, PARP fragmentation, in colon cancer cells (Figure 4b). To determine whether combinatorial therapy induces apoptosis in vivo, we investigated the apoptotic rate using PARP and a terminal deoxynucleotidyl transferase-mediated dUTP nick-154 end labeling (TUNEL) assay. Apoptotic cell death was increased upon combinatorial treatment by 2 assays (Figure 4c,d). We also investigated the relationship between the reactive oxygen species (ROS) and TTFields-induced apoptosis by TTFields in colon cancer cells. ROS level was significantly increased more by combination treatment with TTFields and 5-FU than by TTFields or 5-FU alone.

### 2.5. Combination Treatment Considerably Inhibited Tumor Cell Motility and Tumor Cell Invasion

**Figure 5:**
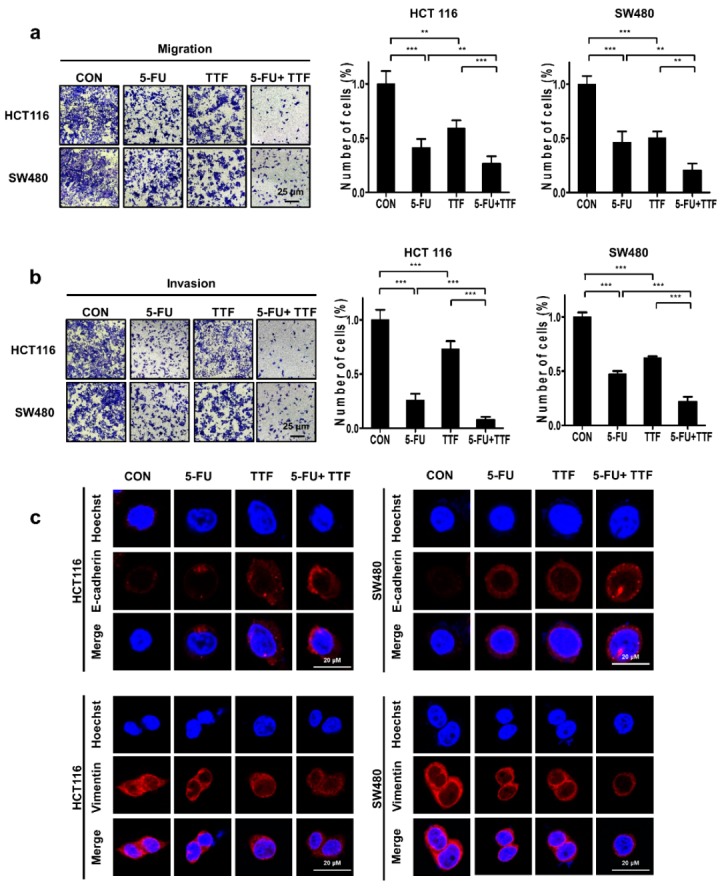
Effect of treatment with 5-FU and TTFields on the migration and invasion of colon cancer cells. (**a**) Tumor cell migration was assessed using the Transwell migration assay; ** *p* < 0.01, *** *p* < 0.001; Values represent the means ± SD (*n* = 3). (**b**) Tumor cell invasion was assayed using the Matrigel invasion assay; *** *p* < 0.001. (**c**) Immunocytochemistry staining of E-cadherin and vimentin expression with and without TTFields treatment or with and without 5-FU treatment.

We next studied the effects of TTFields on the invasive and migratory abilities of colon cancer cells by performing Transwell chamber-migration/ invasion assays. Our experimental results showed that TTFields alone inhibited cell migration. To further investigate the antimetastatic effect of TTFields + 5-FU combination treatment, the migration of 5-FU-treated colon cancer cells was assessed by performing Transwell assays. The results from the Transwell invasion assays showed that invasion capacities after combination treatment gradually decreased compared with that observed with cells treated using 5-FU or TTFields alone (Figure 5a,b).

As shown in these figures, 5-FU, in combination with TTFields significantly inhibited the migration and invasion of both colon cancer cell lines in chamber Transwell assays. We examined EMT biomarkers in TTFields plus 5FU-treated colon cancer cells by immunofluorescence (IF) analysis. EMT is critical for regulating the metastatic potentials [18]. Our results show that TTFields + 5-FU increased the epithelial marker E-cadherin and decreased the expression of the mesenchymal marker, vimentin, compared with single-treatment (Figure 5c). These results suggest that combination treatment with 5-FU and TTFields affected the potentials for cell migration and invasion.

### 2.6. Effects of 5-FU and TTFields on Autophagic Cell Death

**Figure 6:**
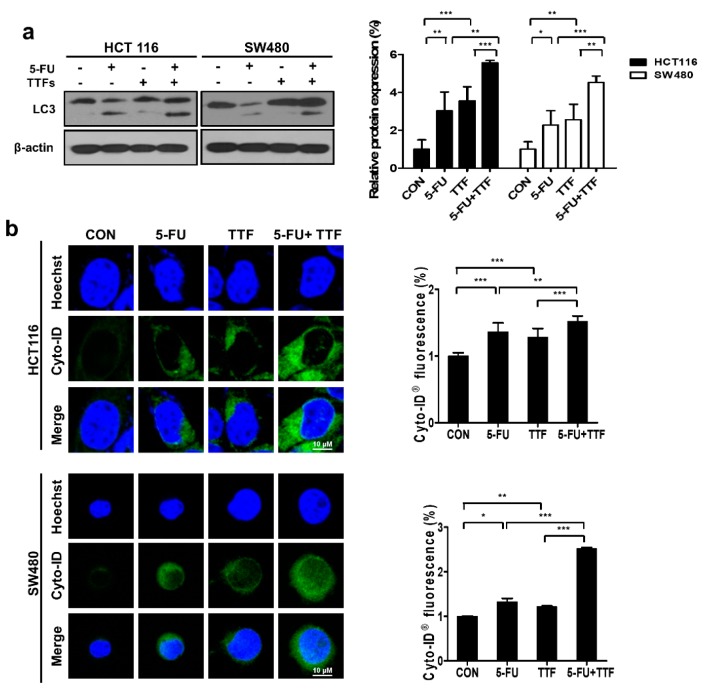

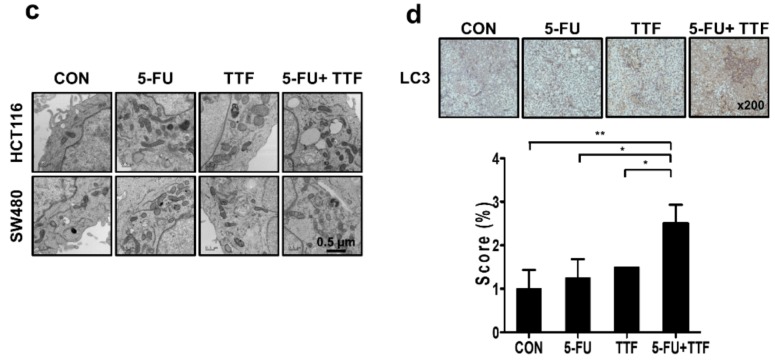
A combination of 5-FU and TTFields induces autophagy in colon cancer cells. (**a**) Cell lysates (30 µg) were immunoblotted (IB) with antibodies against LC3 and β-actin. The relative protein quantification for LC3 was quantified the ratio L3II/LC3I; The ratio was normalized with β-actin; values represent the means ± SD (*n* = 4); * *p* < 0.05, ** *p* < 0.01, *** *p* < 0.001. (**b**) HCT116 and SW480 cell Cyto-ID staining with and without 5-FU, or with and without TTFields treatment. Cells were treated with 5-FU, and TTFields treatment increased the CYTO-ID^®^ dye signal in colon cancer cells; values represent the means ± SD (*n* = 5). * *p* < 0.05, ** *p* < 0.01, *** *p* < 0.001. (**c**) Autophagy measured by transmission electron microscopy in cells. Representative images are provided as indicated. (**d**) LC3 expression in xenografts was determined by immunohistochemistry; values represent the means ± SD (*n* = 3). * *p* < 0.05, ** *p* < 0.01.

To further study the anticancer effects of 5-FU and TTFields, we investigated other cellular responses (autophagy in particular) associated with cell death after 5-FU or TTFields treatment. The expression of LC3 protein was determined (Figure 6a). Western blot analysis (an upper band of 18 kDa for LC3-bI and a lower band of 16 kDa for LC3-bII) showed that autophagosome-lysosome fusion increased with the indicated time after combination treatment. As shown in Figure 6b, there was increased accumulation of Cyto-ID Green around combination-treated HCT116 and SW480 cells. Moreover, transmission electron microscopy was used to prove autophagosome formation in combination-treated cells (Figure 6c). As shown in Figure 6c, combination treatment showed accumulation of large autophagic vacuoles with a typical double-layer membrane and organelle remnants, whereas only a few vacuoles were observed in cells with individual treatment. Furthermore, in vivo mouse xenografts were stained for LC3, to confirm whether the combination treatment could induce greater autophagy than single treatments (Figure 6e). Collectively, our data showed that autophagy was involved in colon cancer cell death after combination treatment in vitro and in vivo.

### 2.7. Combination of 5-FU and TTFs Augmented Cell Death in Colon Patient Organoid

**Figure 7:**
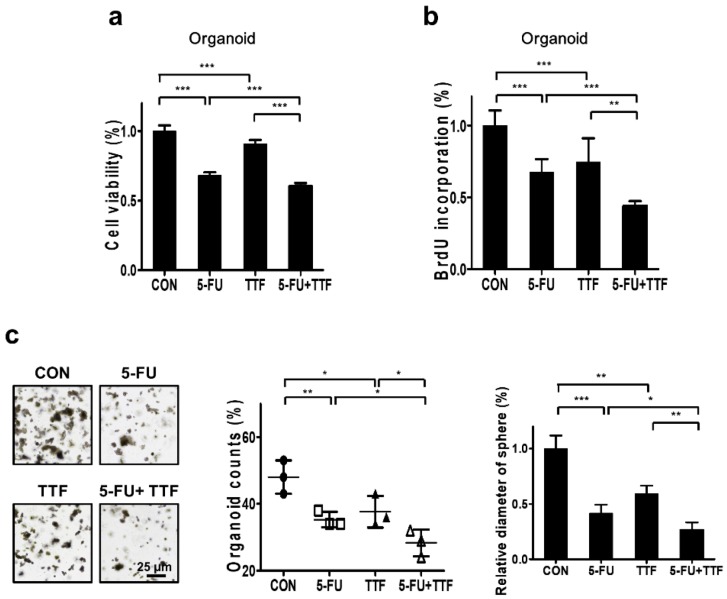

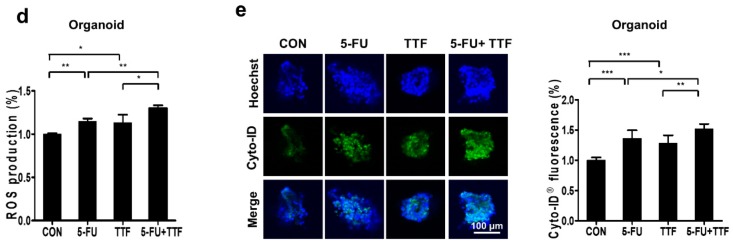
Effect of combinatorial treatment with 5-FU and TTFields on colon patient organoids. Combinatorial treatment inhibited cell proliferation in colon patient organoids. Cell viability and proliferation rate was evaluated by (**a**) MTT assay; *** *p* < 0.001, (**b**) BrdU-labeling; ** *p* < 0.01, *** *p* < 0.001, and (**c**) 3D colony culture; * *p* < 0.05, ** *p* < 0.01, *** *p* < 0.001. (**d**) ROS production was determined using DCF-DA; * *p* < 0.05, ** *p* < 0.01. (**e**) Cyto-ID staining of organoids was detected using confocal laser fluorescence microscopy; * *p* < 0.05, ** *p* < 0.01, *** *p* < 0.001.

To investigate the biological effects of 5-FU and TTFields on colon cell lines, we performed MTT assay and BrdU-labeling (Figure 7a,b). The combination of 5-FU and TTFields treatment had considerably greater antitumor effect on the organoid than either treatment alone. Additionally, the spheres formed by 3D cultures were larger than those formed upon combinatorial treatment (Figure 7c).

We also examined the relationship between ROS level and enhancement in TTFields-induced apoptosis by 5-FU. ROS level was more significantly induced upon combination treatment than upon individual treatments (Figure 7d), which may describe the increase in apoptotic rate upon combination treatment. As shown in Figure 7e, induced accumulation of Cyto-ID Green was shown in combination-treated organoid cells. Our result is consistent with our previous data that suggest its potential as a sensitizer in CRC.

## 3. Discussion

The purpose of this study was to investigate the mechanism by which 5-FU sensitizes CRC cells to TTFields by using in vitro, in vivo, and organoid models. Many researchers reported each cell lines had different sensitivity against 5-FU [19,20] HCT cells were reported as a 5-FU sensitive cell line, and SW480 cells were reported the least 5-FU sensitive cell line. In this study, we showed the TTFiedls-sensitizing effect with 5-FU using HCT116 and SW480 cells to clarify its efficacy, regardless cell lines. We also confirmed using HT29 and SW620 cell lines which were identified modest response against 5-FU [21]. Although, there is a difference the 5-FU concentration between in vitro model and clinical trial, our results demonstrated the effectiveness of TTFields as a sensitizer of 5-FU.

Various clinical trials have been started to estimate the use of 5-FU and combination therapy [22,23]. TTFields has also been undergoing clinical trials for treating many tumor types [13]. Moreover, in the future, TTFields applications should continue to expand in scope to include several new and previously unstudied tumors since there is no severe side effect compared with those of other cancer treatment approaches. Currently, preclinical studies designed to investigate the use of TTFields are underway with several types of cancer [13]. These preclinical studies and subsequent clinical trials will assist in deciding the validity of using TTFields more universally, and thus, we need to support the scientific rationale, investigating the biofunctional mechanisms with TTFields combination therapy for various cancers. The efficacy of TTFields in treating GBM has resulted in FDA approval for utilization in patients with both newly diagnosed and recurrent disease. Given that the targets of TTFields are generic and primarily tumor-type nonspecific, TTFields may offer advantages in various other cancers including GBM. To study that clinical probability research is ongoing to investigate TTFields in many other solid tumors, including colorectal, pancreatic, ovarian, non-small cell lung carcinoma (NSCLC), brain metastases from NSCLC, and malignant mesothelioma for future cancer therapy [24,25,26,27,28]. A recent TTFields study shows that it may also block DNA damage repair, cellular migration, and invasion [29], and regulate autophagy [30]. The resulting daughter cells showed diverse types of cell death, including immunogenic cell death, suggesting that combination of TTFields with immunotherapies may induce antitumor immunity [31]. Moreover, our results on TTFields suggest critical points about the mechanisms by which they influence macrophage-specific immune responses and the efficacy of this protocol for cancer therapy [32]. In preclinical studies, increased sensitivity to chemotherapy in combination with TTFields, was exhibited in human glioblastoma cell lines and animal tumor models [33,34]. A synergistic effect between TTFields and chemotherapy or RT (Radiotherapy) was also reported, suggesting that patients with CRC may derive benefits with this combination in clinical trials [10]. However, the mechanism underlying the enhanced sensitivity to TTFields appears to be slightly more complicated than expected. In our study, we suggested scientific rationales for clinical treatment with 5-FU as a TTFields-sensitizer in CRC. Based on our results, we propose that 5-FU considerably increases the therapeutic efficacy of TTFields by blocking tumor cell survival, cell cycle regulation, and tumor cell invasiveness, while promoting apoptosis and autophagic cell death in human colon cancer cell lines and organoid models. 5-FU, in combination with TTFields decreased clonogenic survival and enhanced the effect of TTFields. TTFields-sensitivity of colon cancer cells promoted apoptosis through increased ROS levels. In RT combination therapy, previous findings have demonstrated that post-radiation 5-FU treatment led to greater radiosensitization than pre-radiation treatment [35]; thus, we need to define this protocol well in TTFields study to apply into clinics effectively. Our mouse and organoid patient data showed that TTFields + 5-FU treatment inhibited tumor growth more than 5-FU treatment alone did. Thus, in future studies, to establish clinical relevance, it is necessary to use an orthotopic model with colon organoid to further study the TTFields-sensitizing effect of 5-FU. In addition, it will be required to compare the sensitizing effects of 5-FU on TTFields for greater efficiency and safety in patients with CRC. In addition, research on the effects of TTFields + 5-FU on normal tissues needs to be performed to lower possible complications in clinical applications for treating patients with CRC.

## 4. Materials and Methods

### 4.1. Experimental Setup for Electric Fields

TTFields was produced using a pair of insulated wires (Seoil Electric Wire Co., Ltd., Eumseong-gun, Chungcheongbuk-do, Korea; outer diameter, 0.4 mm; polyvinyl chloride insulation thickness, 0.17 mm; dielectric breakdown, 25 kV/mm) connected to a function generator (AFG-2112, Good Will Instrument Co., Ltd., New Taipei City, Taiwan) and a high-voltage amplifier (A303, A. A. Lab Systems, Ltd., Ramat-Gan, Israel) that generated sine-wave signals ranging from 0 to 800 V [36].

### 4.2. Antibodies and Chemicals

Antibodies recognizing cleaved PARP (Asp214) was purchased from Cell Signaling Technology (Danvers, MA, USA) and Novos Biologicals, LLC (Centennial, CO, USA). LC3A/B was purchased from Cell Signaling Technology, and a beta-actin antibody was purchased from Santa Cruz Biotechnology (Dallas, TX, USA). Dulbecco’s modified Eagle’s medium (DMEM), fetal bovine serum (FBS), penicillin-streptomycin, and phosphate-buffered saline (PBS) were purchased from WELGENE, Inc. (Gyeongsan-si, Gyeongsangbuk-do, Korea). Trypsin–EDTA was purchased from Gibco (Gaithersburg, MD, USA). 5-FU was purchased from Abcam (Cambridge, UK). For in vitro experiments, 5-FU was dissolved in dimethyl sulfoxide to prepare a 50 mM stock solution, which was stored at 4 °C.

### 4.3. Cell Culture

HCT116 and SW480 CRC cell lines were obtained from the American Type Culture Collection (Manassas, VA, USA). HT29 and SW620 cell lines were kindly provided by Dr. Youn Kyoung Jeong and Jong Kuk Park in Korea Institute of Radiological and Medical Sciences (KIRAMS). The cells were grown in DMEM supplemented with 10% FBS, l-glutamine, 4-(2-hydroxyethyl)-1-piperazineethanesulfonic acid (HEPES), and antibiotics at 37 °C in a humidified incubator under 5% CO_2_.

The organoids were obtained from Dr. Shin at the Korea Cancer Center Hospital. The patient samples were collected by surgical resection or endoscopic biopsy at Korea Cancer Center Hospital after receiving informed consent, the study was approved by the ethical committee of the hospital (No. KIRAMS-2017-07-001, Approval Date: 09/04/2017). All procedures performed in studies involving human participants were in accordance with the ethical standards of the institutional and/or national research committee.

Tumor tissues were isolated from the tissues, as described previously, with some minor modifications. In brief, cancer tissues were incubated in collagenase type II (Sigma-Aldrich, St. Louis, MO, USA), dispase type II (Roche Applied Science, Mannheim, Germany), and Y-27632 (BioVision, Mountain View, CA, USA) for 30 min at 37 °C. The CRC-PDO culture medium contained 1× B27 (Gibco, Grand Island, NY, USA), 1.25 mM N-acetyl cysteine (United States Pharmacopeia, Rockville, MD, USA), 50 ng/mL human epidermal growth factor (BioVision, Mountain View, CA, USA), 50 ng/mL human Noggin (Peprotech, Rocky Hill, NJ, USA), 10 nM gastrin (Sigma-Aldrich), 500 nM A83-01 (BioVision, Mountain View, CA, USA), and 100 mg/mL primocin (InvivoGen, San Diego, CA, USA). During the first 2–3 days, 10 μM Y-27632 was added to the culture medium to prevent anoikis. The organoids were passaged every week by pipetting using Gentle Cell Dissociation Reagent (STEMCELL Technologies, Vancouver, BC, USA).

### 4.4. Cell Viability Assay

Cells were seeded at a density of 5,000 cells per well in a 96-well plate and incubated for 24 h. EZ-CYTOX (DAEILLAB SERVICE Co, Ltd., Seoul, Korea) was added to cells after 5-FU treatment. The 5-FU was treated according to the indicated experimental conditions as described previously [19]

4.5. 3D Culture System

Human HCT116 and SW480 cells were seeded in 96-well plates at 1 × 10^4^ cells/well according to the manufacturer’s protocol [19]

### 4.6. Colony-Forming Assay

TTFields was applied to cells 6 h after 5-FU exposure to a final concentration of 5 μmol/L, and the cells were then incubated for 48 h. After 10–12 days, colonies were stained with 0.4% crystal violet (Sigma, St. Louis, MO, USA) according to the manufacturer’s protocol [19]

### 4.7. Tumor Xenografts in Nude Mice

A single-cell suspension (4 × 10^6^ cells) was subcutaneously injected into the flanks of 6-week-old BALB/c nude mice (Orient Bio Co., Ltd.; Gyeonggi-do, Korea). The product was approved by the Institutional Animal Care and Use Committee of KIRAMS (kirams2019-0073, 28/06/2019). When each tumor reached a minimal volume of 500 to 100 mm^3^, 1 V/cm TTFields, 30 mg/kg 5-FU (3 times a week), or combination treatment was started and applied for 7 days. Tumor volumes were determined according to the formula (Length × Width^2^) × 3.14/6 by measuring tumor length (L) and width (*l*) with a caliper. All animal work was conducted in accordance with our institute’s policies.

### 4.8. PET/CT Scans and Image Analysis

A small animal PET/CT scanner (nanoScan^®^, Mediso Medical Imaging Systems, Budapest, Hungary) was used for obtaining whole-body PET images of mice. Before FDG uptake, the mice were warmed using a heating pad. Then, 7.8 ± 0.6 MBq of [^18^F]-FDG was injected into the tail vein, and the mice were anesthetized with 2% isoflurane in 100% oxygen (Forane solution; ChoongWae Pharma, Seoul, Korea). A 20 min static acquisition was started 40 min after [^18^F]-FDG injection. The scanner has a peak absolute system sensitivity of >9% in the 250–750 keV energy window, an axial field of view of 28 cm, a transaxial field of view of 35–120 mm, and a transaxial resolution of 0.7 mm at 1 cm off-center. PET data were reconstructed using voxel dimensions of 0.86 × 0.86 × 0.80 mm^3^ by a 3-dimensional order-subset expectation-maximization algorithm (4 iterations and 6 subsets). For attenuation correction, micro-CT imaging was conducted immediately after PET using 50 kVp of X-ray voltage with 0.16 mAs.

After co-registration of the CT and PET data, volumes of interest (VOIs) were manually defined for the tumor. The maximum pixel values within the ROI on the PET image were then measured and normalized in units of standardized uptake value (SUV), which is calculated to normalize for differences in injected dose and body weight. The SUV values obtained for each region of activity were multiplied by the mouse weight divided by the injected dose.

### 4.9. Detection of Apoptotic Cells via Annexin V Staining

After 5-FU exposure, TTFields was applied to the cells, which were then incubated for a further 48 h. The apoptotic cells were evaluated by using an Annexin V-FITC/PI apoptosis detection kit (BD Biosciences, San Jose, CA, USA) according to the manufacturer’s protocol [19]

### 4.10. Western Blotting

After 5-FU treatment, the colon cancer cells were exposed to TTFields, which were then incubated for 24 or 48 h. The cells were then lysed with RIPA buffer; proteins were separated by sodium dodecyl sulfate-polyacrylamide gel electrophoresis and transferred to nitrocellulose membranes according to the manufacturer’s protocol [14]

### 4.11. TUNEL Assays

The apoptotic cells were confirmed in situ using an apoptosis detection kit (Millipore, Burlington, MA, USA). Tumors were collected and fixed with 10% neutral-buffered formalin and then, the assay was performed according to the manufacturer’s protocol [25]

### 4.12. Fluorescence-Based Quantification of Intracellular ROS

The cellular reactive oxygen species detection assay kit (Abcam, Cambridge, UK) was used to assess intracellular ROS production. For fluorocytometric analysis, HCT116 and SW480 cells were treated for 24 h with TTFields, 5-FU, or a combination of both, and ROS were detected according to the manufacturer’s protocol [19].

### 4.13. Cyto-ID^®^ Analysis

Cells were treated, harvested, and stained with the Cyto-ID^®^ Green Detection Reagent and Hoechst 33342 according to the manufacturer’s protocol [37]. The Cyto-ID^®^ Autophagy Detection Kit 2.0 (Enzo Biochem, Inc., New York, NY, USA) was then used and cells were visualized with a confocal laser scanning microscope (LSM 880, Carl Zeiss Co. Ltd., Oberkochen, Germany).

### 4.14. Transmission Electron Microscopy

HCT116 and SW480 cells were treated for 48 h with TTFields, 5-FU, or both in combination, and then fixed in 2.5% glutaraldehyde (Sigma-Aldrich, St. Louis, MO, USA). A Sorvall MT5000 microtome (DuPont Instruments, Wilmington, USA) was used to prepare ultrathin sections after dehydration. Lead citrate and/or 1% uranyl acetate was used to stain the sections, and the autophagic vacuoles in the cytoplasmic area were calculated using Image Pro Plus software, version 3 (Media Cybernetics, Inc., Silver Spring, MD, USA).

### 4.15. Immunohistochemistry

For immunohistochemical assessments, 4-μm paraffin-embedded CRC sections were mounted on coated glass slides to detect the proteins under investigation. Following antigen retrieval and blocking of endogenous peroxidases and nonspecific protein binding, slide sections were incubated with primary antibodies, followed by horseradish peroxidase-conjugated secondary antibodies. The primary antibodies for Ki-67 and LC3 (diluted 1:200) were purchased from Cell Signaling Technology. All slides were developed with 3,3’-diaminobenzidine, followed by hematoxylin counterstaining.

### 4.16. Invasion/Migration Assay

Invasion and migration abilities were measured in vitro using Transwell chambers, as described previously [14]

### 4.19. Statistical Analysis

All data are presented as means ± SD. Statistical analysis of in vitro data sets was performed with GraphPad Prism 5 software (GraphPad Inc., San Diego, CA, USA) using unpaired one-tailed student’s *t*-test. Data sets of in vivo were analyzed by two-way ANOVA. *p* < 0.05 was considered to indicate statistical significance.

### 4.20. Ethical Statement

The study was approved by the ethical committee of the hospital (No. KIRAMS-2017-07-001, KIRAMS 2019-0073). All procedures performed in studies involving human participants or animals were in accordance with the ethical standards of the institutional and/or national research committee.

## 5. Conclusions

In conclusion, combination treatment with 5-FU and TTFields enhanced the efficacy of TTFields therapy in colon cancer cells by downregulating signaling pathways involved in cell proliferation/survival, cell invasion, and migration, while upregulating apoptosis and autophagic cell death. Our results based on in vitro, in vivo experiments, and human organoids suggest that TTFields may cause selective damage to cancer tissues, further proving the potential of TTFields plus 5-FU as a better alternative to conventional cancer treatment protocols.

## Figures and Tables

**Table 1:** Evaluation of the TTFields effect of 5-FU according to the formulas of Valeriote and Carpentier.

Cell Type	TTFields (V/cm)	SF_TTF + 5-FU_	SF_TTF_ × SF_5-FU_	TTF Effect
HCT116	0.9	0.950	0.953	Synergism
	1.2	0.811	0.926	Synergism
	1.5	0.653	0.824	Synergism
SW480	0.9	0.201	0.421	Synergism
	1.2	0.089	0.141	Synergism
	1.5	0.084	0.113	Synergism

(SF: surviving fraction)

## Supplementary Materials

The following are available online at https://www.mdpi.com/2072-6694/11/12/1999/s1, Figure S1: Blood test results for TTFields and 5-FU treatment of the colon in vivo models. Figure S2: TTFields-sensitizing effect with 5-FU in HT29 and SW620 cell lines.

## Abbreviations

3D	Three dimensional
5-FU	5-Fluorouracil
CRC	Colorectal cancer
CT	Computed tomography
DCFH-DA	2’,7’-Dichlorofluorescein diacetate
DMEM	Dulbecco’s modified Eagle’s medium
FBS	Fetal bovine serum
FDG	^18^F-fluoro-2-deoxy-d-glucose
GBM	Glioblastoma multiforme
HEPES	4-(2-Hydroxyethyl)-1-piperazineethanesulfonic acid
IR	Infrared radiation
MTT	3-(4,5-Dimethylthiazol-2-yl)-2,5-diphenyltetrazolium bromide
NSCLC	Non-small cell lung carcinoma
PBS	Phosphate buffered saline
PE	Plating efficiency
PET	Positron emission tomography
PI	Propidium iodide
ROI	Region of interest
ROS	Reactive oxygen species
SD	Standard deviation
SUV	Standard uptake value
TTFields	Tumor-treating fields
TUNEL	Terminal deoxynucleotidyl transferase-mediated dUTP nick-end labeling
RT	Radiotherapy
SF	Surviving fraction
