# Supplementary material for: 5-Fluorouracil as a Tumor-Treating Field-Sensitizer in Colon Cancer Therapy
Source: Cancers (Basel). 2019 Dec 12;11(12):1999. doi: 10.3390/cancers11121999 (PMC6966590; doi:10.3390/cancers11121999)
Supplement: Supplementary file 1 [file cancers-11-01999-s001.zip › cancers-632528-suppl-final/cancers-632528-Suppl-final.docx]

Article

5-Fluorouracil as a Tumor-Treating Field-Sensitizer in Colon Cancer Therapy

Yeon-Joo Lee ^1^, Jae-Min Cho ^1^, Sei Sai ^2^, Ju Yeon Oh ^3^, Ji-Ae Park ^4^, Se Jong Oh ^4^, Misun Park ^5^, Junhye Kwon ^5^, Ui Sup Shin ^6^, Jeong-Hwa Beak ^7^, Sun Ha Lim ^8^_,_ Jie-Young Song ^1^_,_ Sang-Gu Hwang ^1,^* and Eun Ho Kim ^8,^*


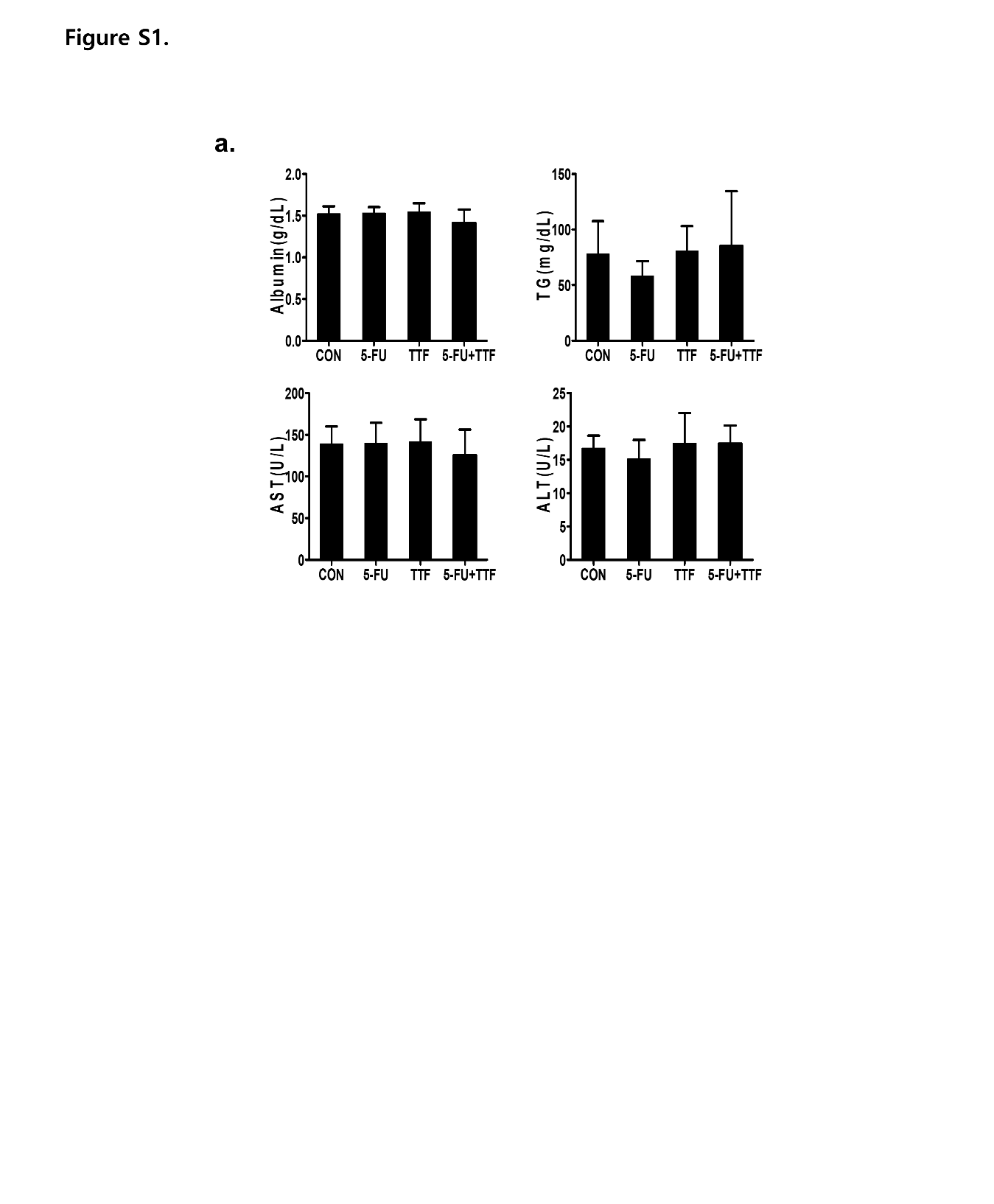


**Figure S1.** Blood test results for TTFields and 5-FU treatment of the colon in vivo models. The blood was collected and tested at the end of the experiment (7 days).


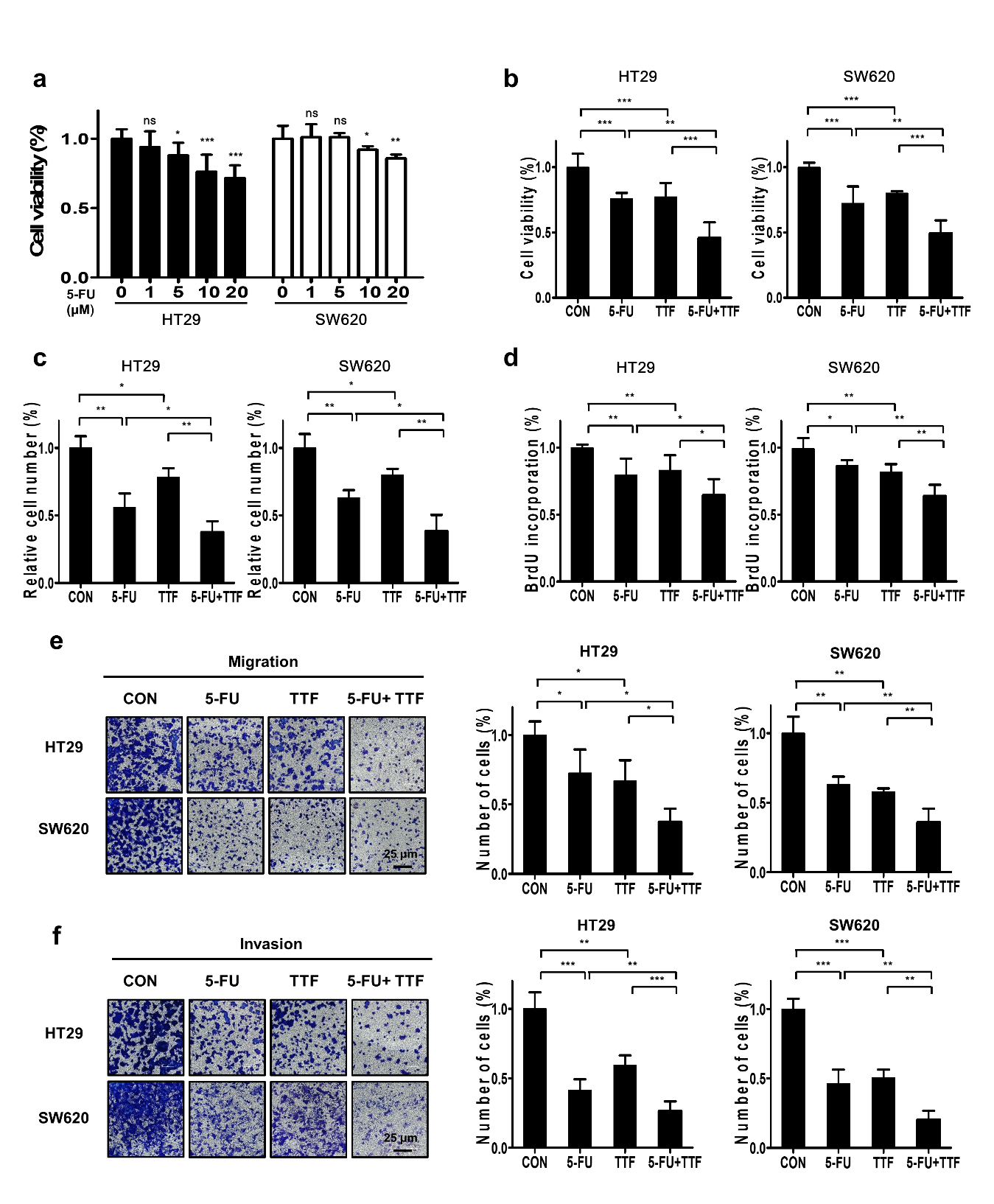


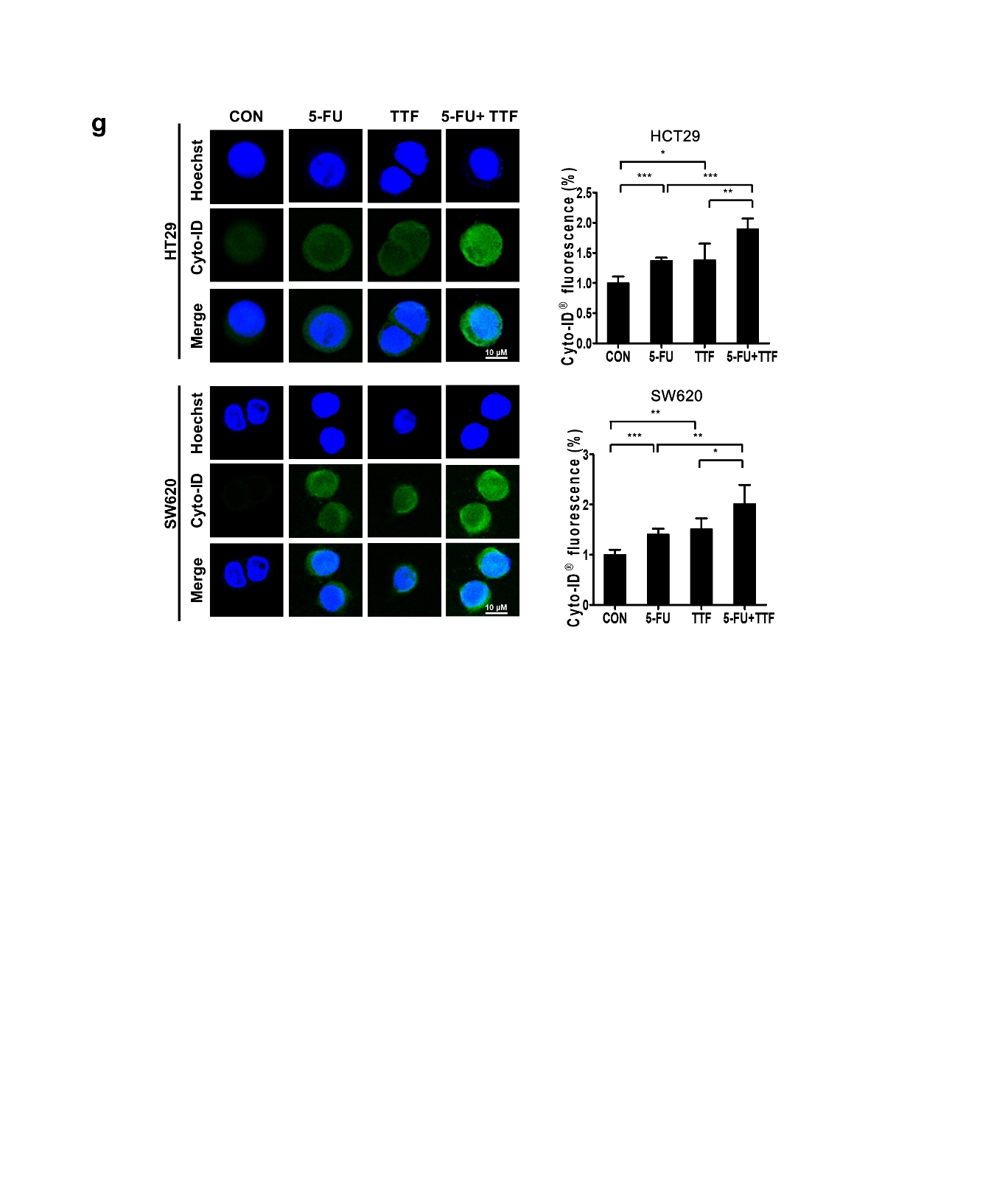


**Figure S2.** TTFields-sensitizing effect with 5-FU in HT29 and SW620 cell lines. (**a**,**b**) The cell viability was measured by MTT assay in HT29 and SW620; ns: not significant; Values represent the means ± SD (*n* = 5); * *p* < 0.05, ** *p* < 0.01, *** *p* < 0.001. (**c**,**d**) The proliferation rate was evaluated by trypan blue cell viability assay (*n* = 3) and BrdU-labeling (*n* = 5); * *p* < 0.05, ** *p* < 0.01. (**e**,**f**) The Transwell migration assay was estimated to assess tumor cell migration and invasion; Values represent the means ± SD (*n* = 3); * *p* < 0.05, ** *p* < 0.01, *** *p* < 0.001. (**g**) Autophagy measured by CYTO-ID^®^ dye signal in colon cancer cells; Values represent the means ± SD (*n* = 3); * *p* < 0.05, ** *p* < 0.01, *** *p* < 0.001.
