# Supplementary material for: 5-Fluorouracil as a Tumor-Treating Field-Sensitizer in Colon Cancer Therapy
Source: Cancers (Basel). 2019 Dec 12;11(12):1999. doi: 10.3390/cancers11121999 (PMC6966590; doi:10.3390/cancers11121999)

Fig.4b

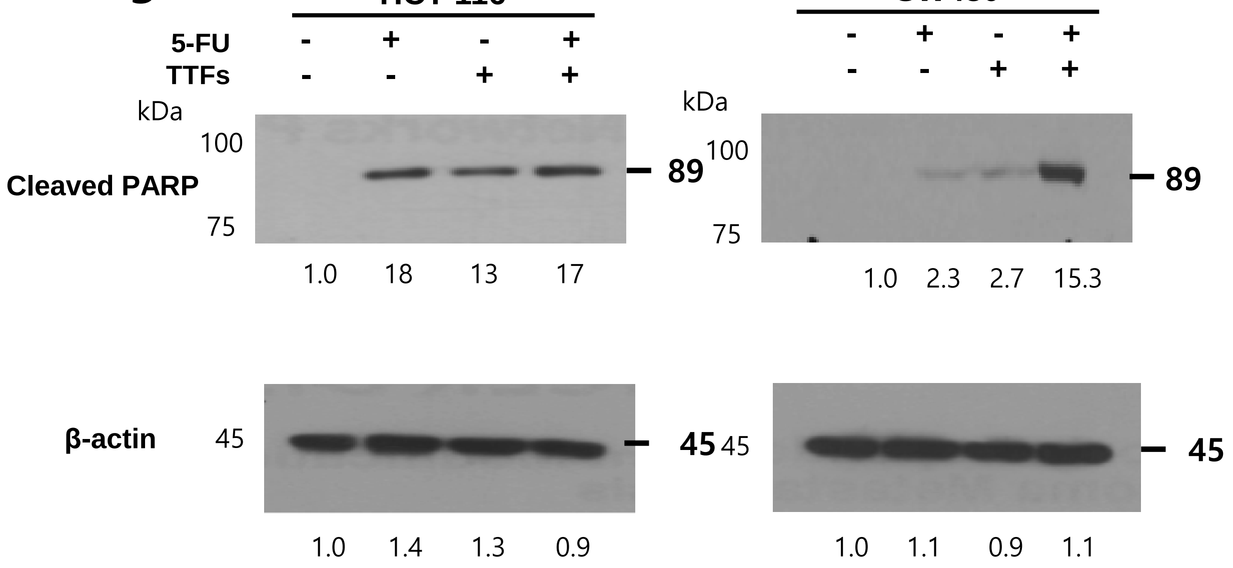
HCT 116
SW480
5-FU
TTFs
kDa
100
75
Cleaved PARP
89
1.0 18 13 17
1.0 2.3 2.7 15.3
β-actin
45
1.0 1.4 1.3 0.9
1.0 1.1 0.9 1.1

Fig.6a

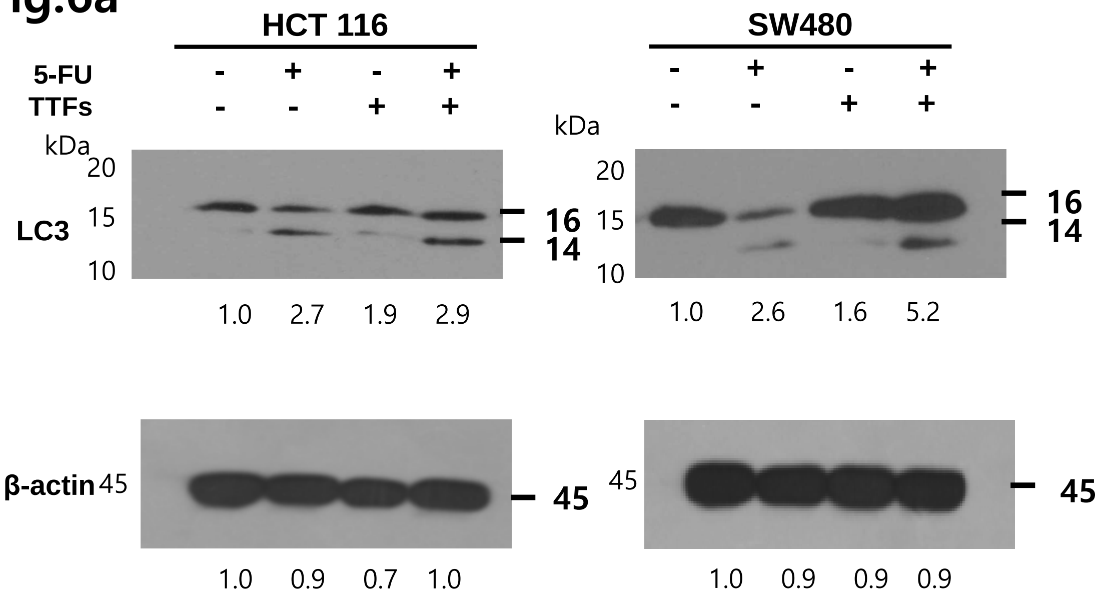
HCT 116
SW480
5-FU
TTFs
kDa
20
15
10
LC3
16
14
1.0 2.7 1.9 2.9
1.0 2.6 1.6 5.2
β-actin
45
1.0 0.9 0.7 1.0
1.0 0.9 0.9 0.9

## Cleaved PARP (89kDa)

### HCT 116

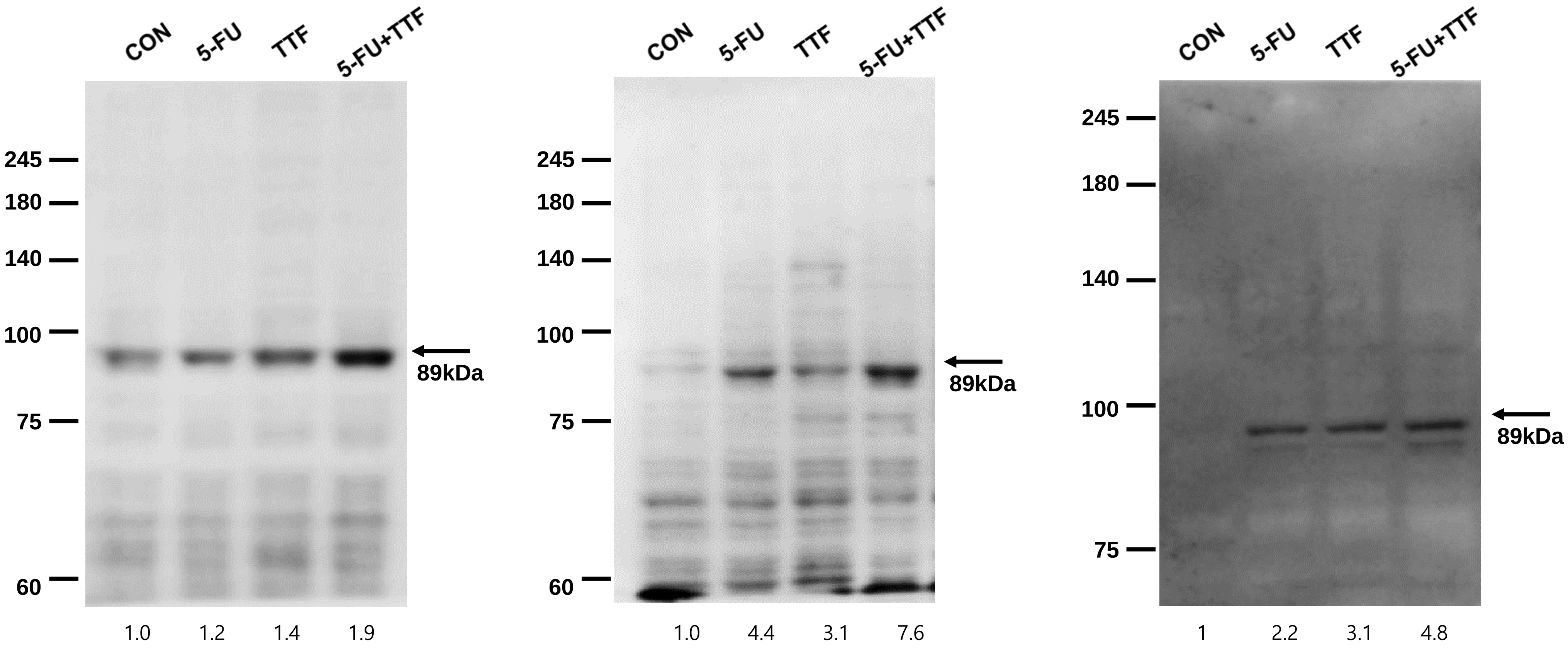

**β- actin (45kDa)**

**HCT 116**

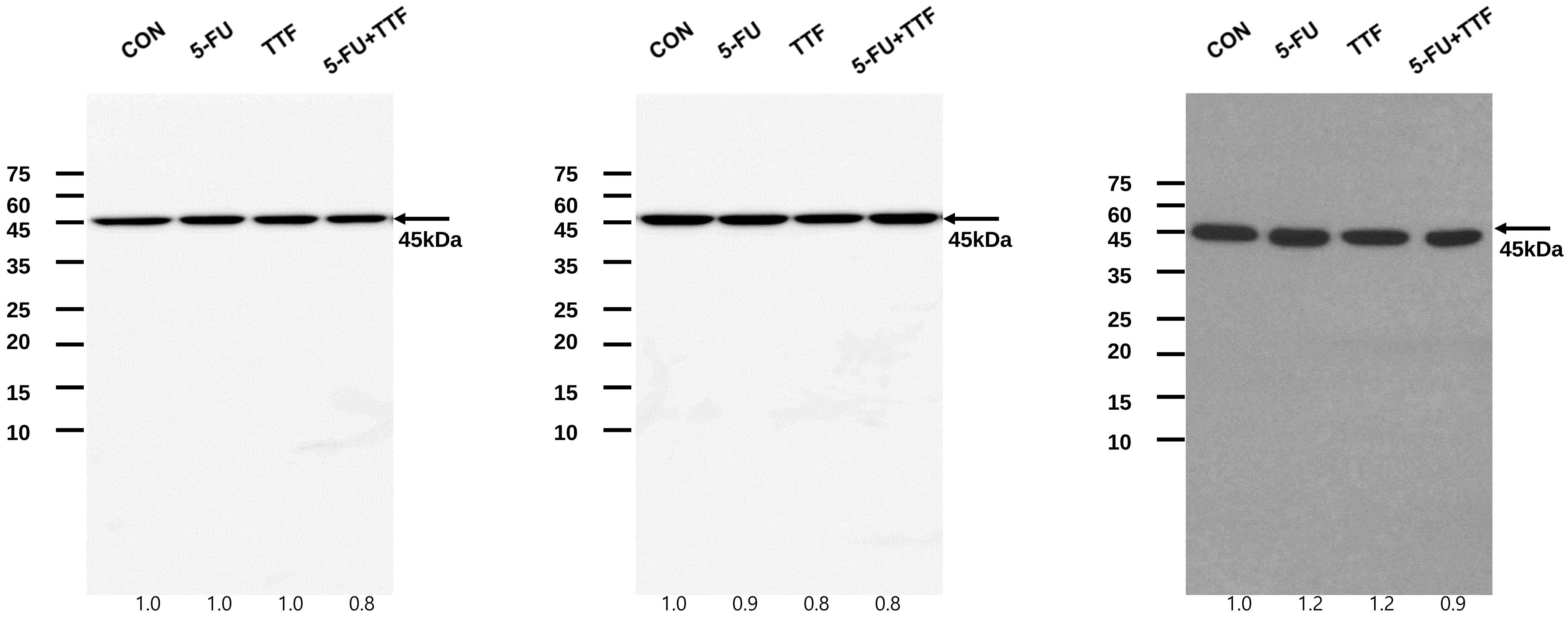

# Cleaved PARP (89kDa)

SW480

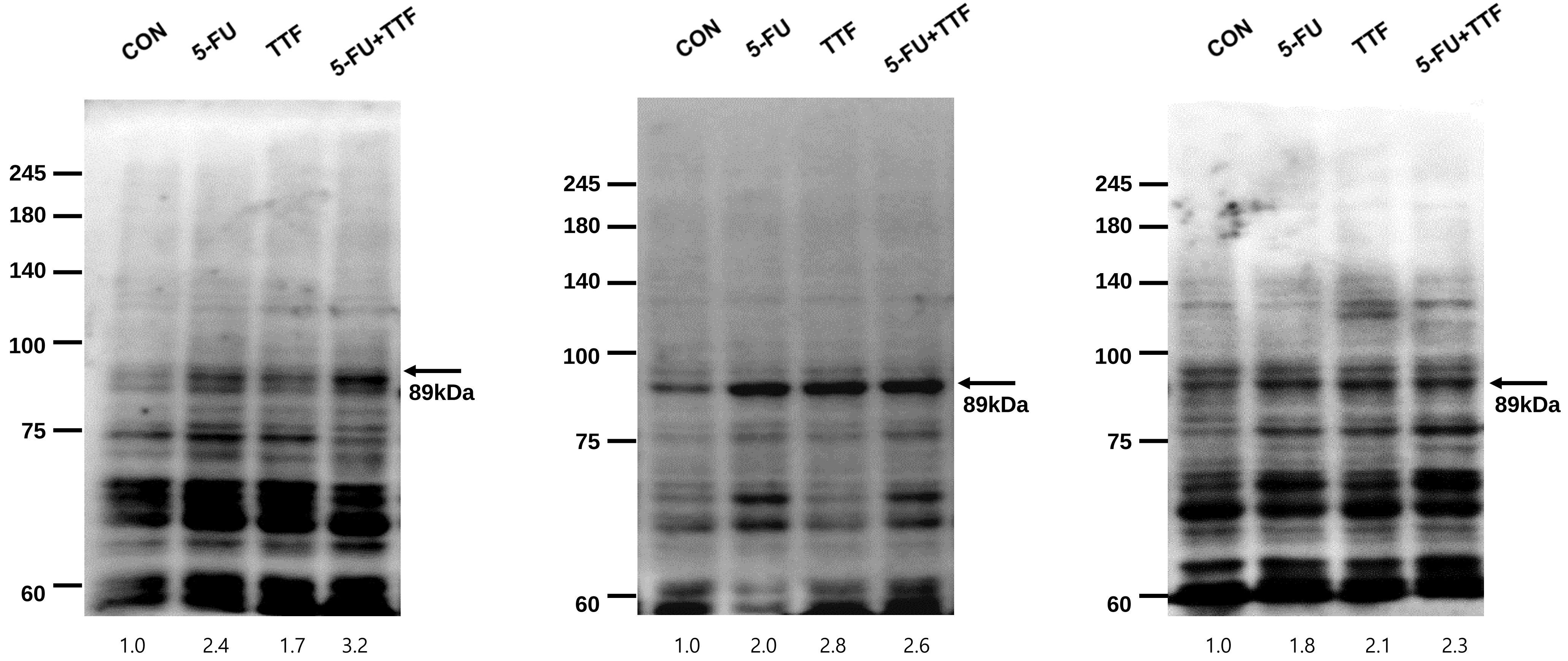

**β- actin (45kDa)**

**SW480**

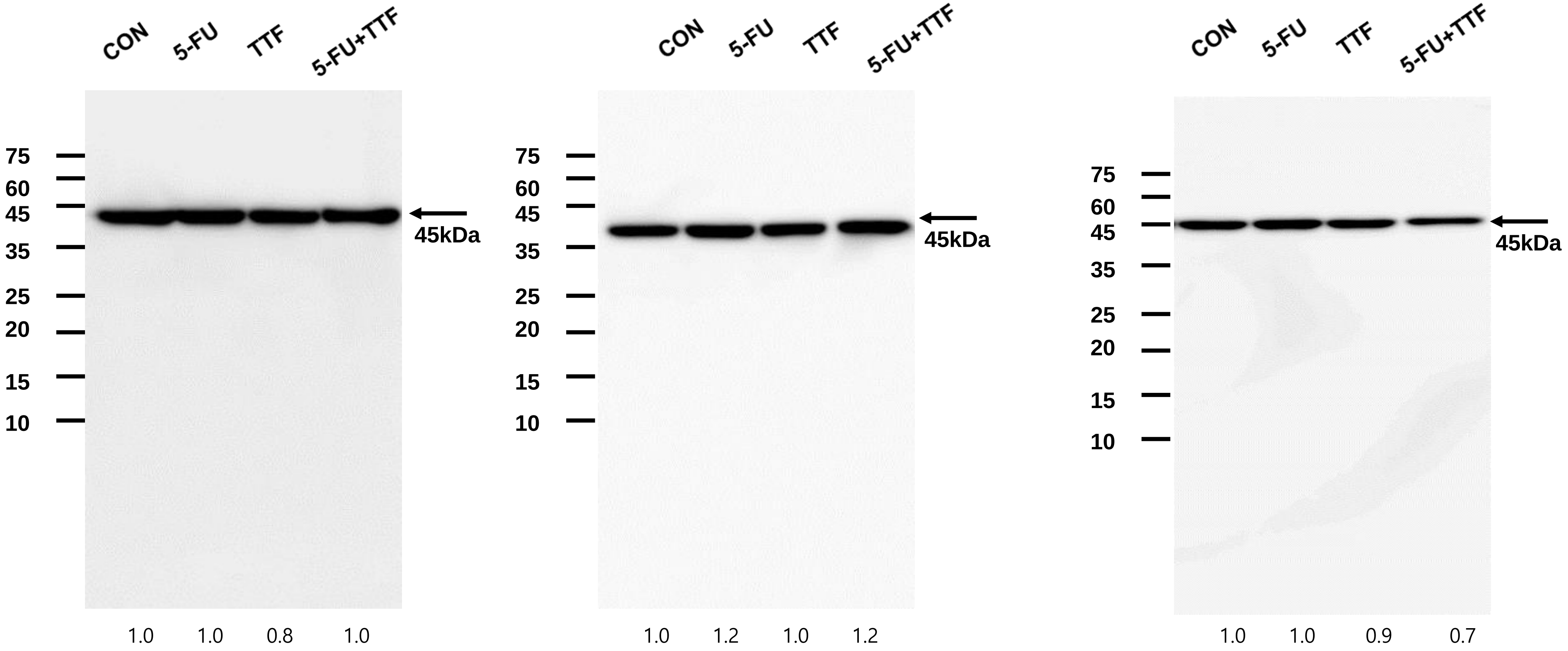

# LC3A/B (14,16 kDa)

**HCT116**

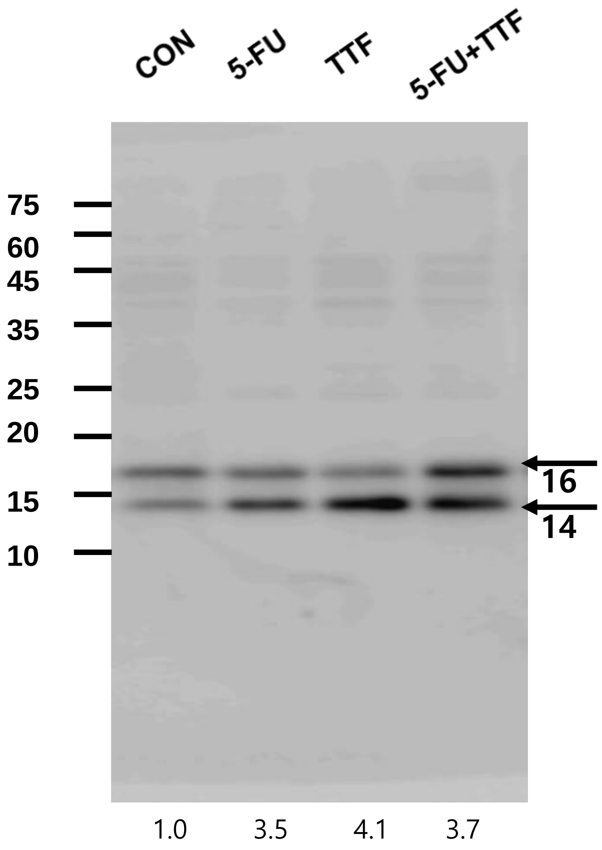


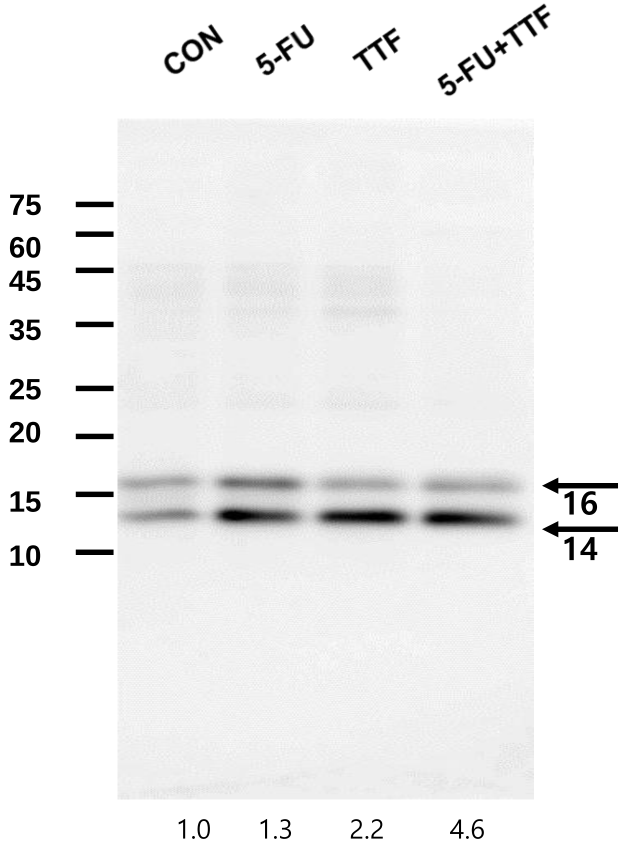


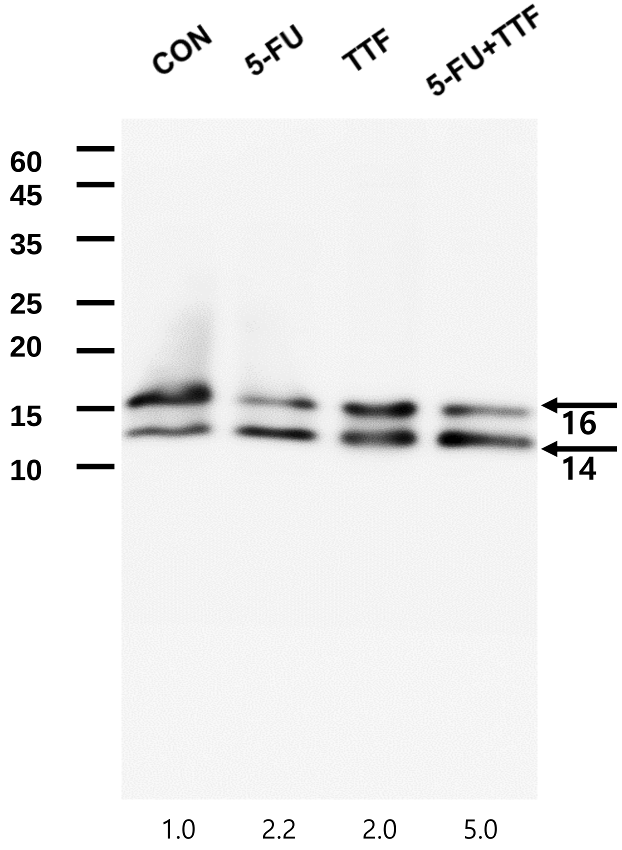

β- actin (45kDa)

HCT116

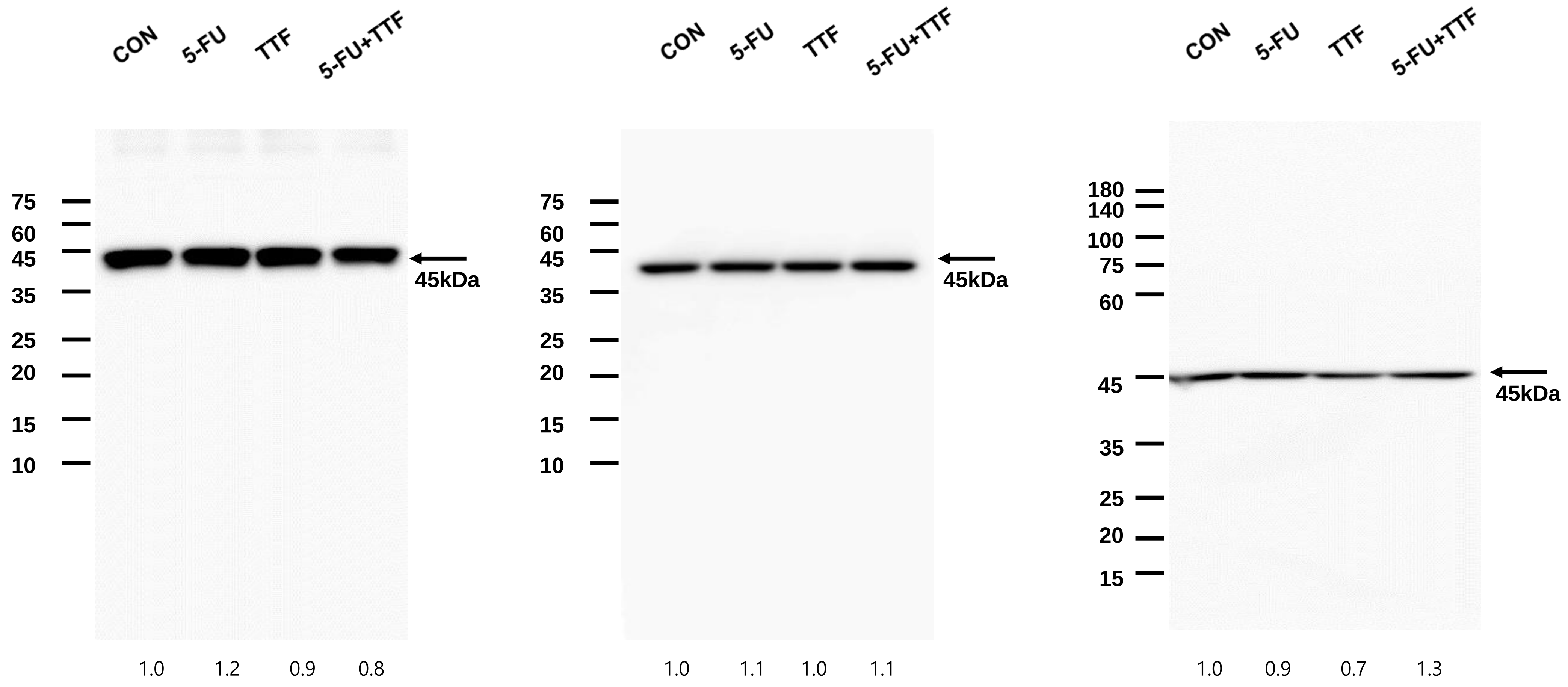

**LC3 (16,14 kDa)**

**SW480**

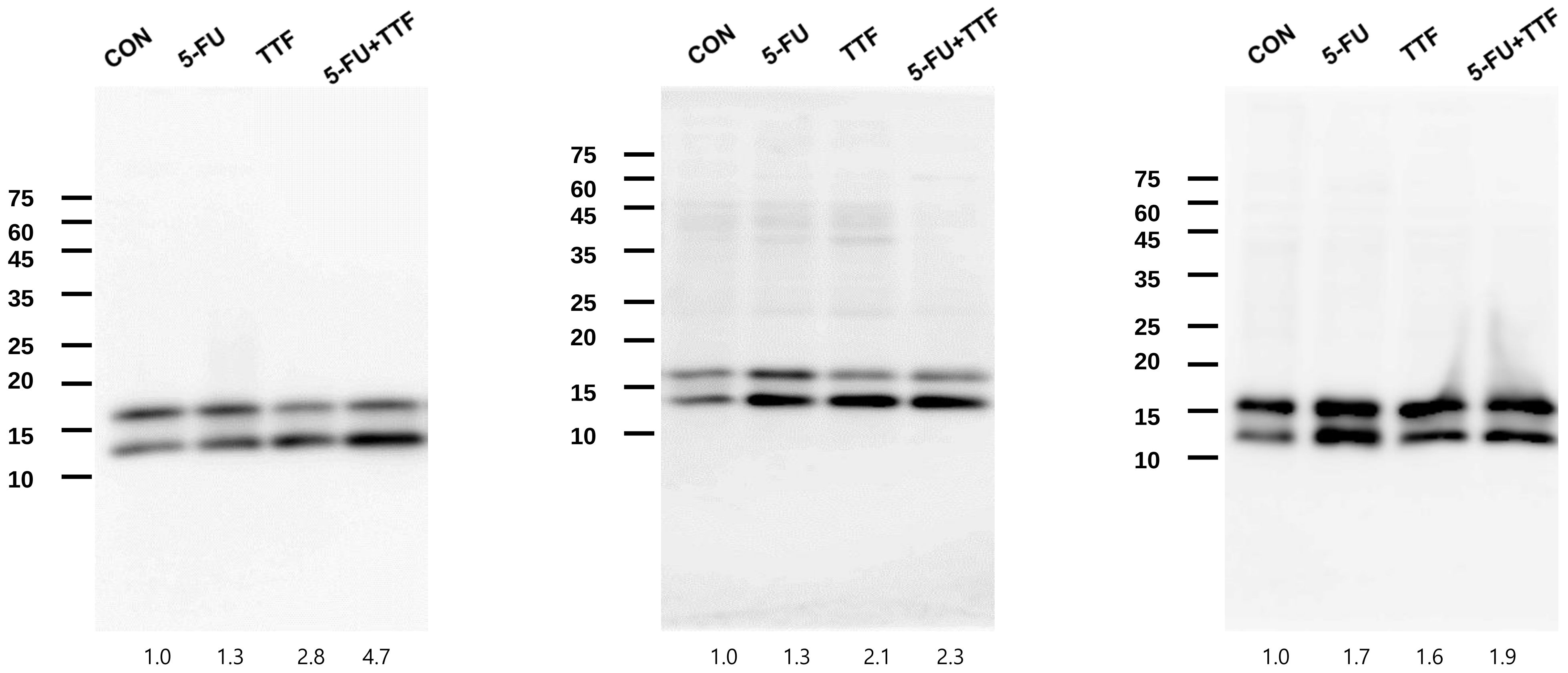

# β- actin (45kDa)

**SW480**

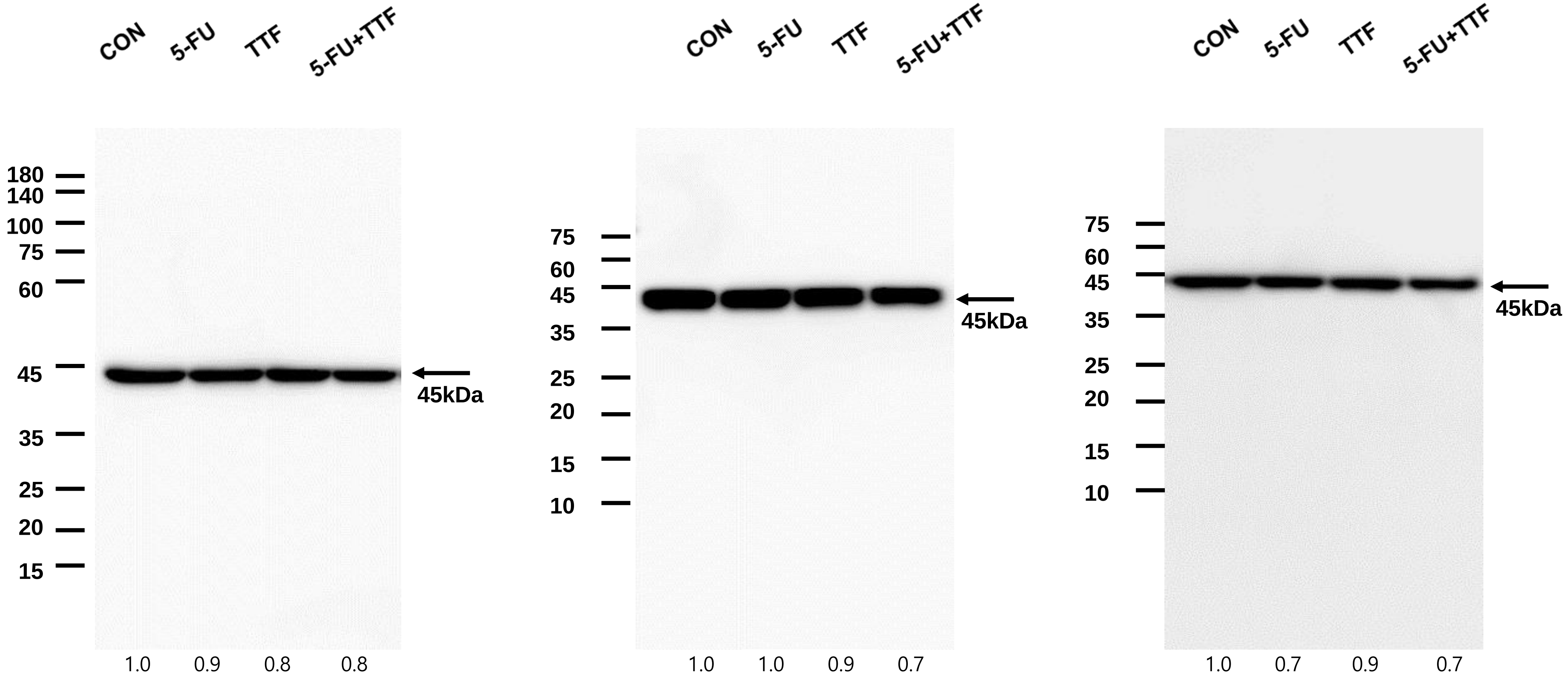

Supplement: Supplementary file 1 [file cancers-11-01999-s001.zip › cancers-632528-suppl-final/cancers-632528-western blot figures.pdf]
